# Mass Spectrometry Quantification of Epigenetic Changes: A Scoping Review for Cancer and Beyond

**DOI:** 10.3390/ijms27010149

**Published:** 2025-12-23

**Authors:** Rossana Comito, Agnese Mannaioli, Agen Peter Lunghi Msemwa, Francesca Bravi, Carlotta Zunarelli, Eva Negri, Emanuele Porru, Francesco Saverio Violante

**Affiliations:** 1 Division of Occupational Medicine, IRCCS Azienda Ospedaliero-Universitaria di Bologna, 40138 Bologna, Italy; rossana.comito@aosp.bo.it (R.C.); agenpeter.lunghimsemwa@aosp.bo.it (A.P.L.M.); carlotta.zunarelli2@unibo.it (C.Z.); francesco.violante@unibo.it (F.S.V.); 2 Department of Clinical Sciences and Community Health, Dipartimento di Eccellenza 2023-2027, University of Milan, 20133 Milan, Italy; francesca.bravi@unimi.it; 3 Occupational Medicine Unit, Department of Medical and Surgical Sciences, Alma Mater Studiorum University of Bologna, 40138 Bologna, Italy; eva.negri@unibo.it

**Keywords:** epigenetic modifications, mass spectrometry, DNA methylation, histone post-translational modifications, cancer biomarker

## Abstract

Mass spectrometry has become an indispensable tool for the identification and quantification of epigenetic modifications, offering both high sensitivity and structural specificity. The two major classes of epigenetic modifications identified—DNA methylation and histone post-translational modifications—play fundamental roles in cancer development, underscoring the relevance of their precise quantification for understanding tumorigenesis and potential therapeutic targeting. In this scoping review, we included 89 studies that met the inclusion criteria for detailed methodological assessment. Among these, we compared pre-treatment workflows, analytical platforms, and acquisition modes employed to characterize epigenetic modifications in human samples and model systems. Our synthesis highlights the predominance of bottom-up strategies combined with Orbitrap-based platforms and data-dependent acquisition for histone post-translational modifications, whereas triple quadrupole mass spectrometers were predominant for DNA methylation quantification. We critically evaluate current limitations, including heterogeneity in validation reporting, insufficient coverage of combinatorial post-translational modifications, and variability in derivatization efficiency.

## 1. Introduction

DNA plays a pivotal role in transmitting all the genetic information required for the correct development, function, growth and reproduction of all known living organisms and many viruses. Consequently, it is fundamental in shaping biological phenotypes and orchestrating the intricate process of life. Changes in its composition, sequence, or structure can cause a variety of physiological dysfunctions. DNA stability and integrity are crucial for proper cellular functioning and the accurate transmission of hereditary traits across generations. Deviations from the normal state of DNA can have serious consequences for an organism’s health and well-being, leading to a wide range of pathologies, including genetic diseases and cancers [1].

Recently, there has been a growing interest in understanding the mechanisms by which DNA modifications arise and how they affect the structure and function of DNA. The final goal is to gain a comprehensive understanding of their broader implications in both normal physiological processes and disease states. Epigenetics is often defined as functionally relevant changes to the genome that occur without altering the nucleotide sequence [2]. Key mechanisms responsible for these phenomena include: DNA methylation, histone modifications, and chromatin remodeling [3]. Techniques such as chromatin immunoprecipitation sequencing (Chip-seq), bisulfite sequencing, methylated DNA immunoprecipitation, and chromosome conformation capture have been instrumental in improving our understanding of the epigenetic regulation of gene expression [4]. In addition, advances in mass spectrometry (MS) technologies have greatly improved the analysis of epigenetic DNA damage and modifications.

Of all the epigenetic modifications, DNA methylation is the most widely studied. DNA methylation patterns are highly dysregulated in cancer. In fact, changes in methylation status have been postulated to inactivate tumor suppressors and activate oncogenes, thus contributing to tumorigenesis [5]. Currently, there are three main groups of techniques that involve the identification of specific regions that are differentially methylated: bisulfite conversion-based methods, restriction enzyme-based approaches, and affinity enrichment-based assays [6].

In addition to noncanonical bases that are actively generated for partially unknown purposes, genomic DNA (gDNA) contains modified bases that are generated as DNA lesions. In particular, oxidative DNA lesions such as 8-oxo-7,8-dihydro-deoxyguanosine (8oxodG) can easily form [7], and the levels of such base lesions can correlate with diseases [8]. Although 5-Methyl-2′-deoxycytidine (5mdC) is the most abundant epigenetic modification, its total content is only around 2%. Following in prevalence is 5-hydroxymethyl-2′-deoxycytidine (5hmdC), typically found at levels below 1%. Other modifications, such as 8oxodG and N6-methyl-2′-deoxyadenosine, are significantly rarer, occurring at approximately 0.001% and 0.0001%, respectively [1].

Histones are basic proteins whose positive charges enable them to combine with DNA. Covalent post-translational modifications (additions or removal of functional groups) mainly occur on side chains of histone tails [9]. Certain modifications alter the charge density between histones and DNA, thereby affecting the organization of the chromatin, modifying its accessibility to the enzymes orchestrating gene transcription [3,10]. Thereby, gene expression is affected by histone modifications [3].

Detecting and quantifying chemical DNA modifications, especially epigenetic alterations, is crucial for disease screening and treatment. Thanks to its high sensitivity, analytical precision, and ability to characterize complex molecular changes, mass spectrometry has become a key tool in the study of epigenetic modifications.

Given the rapid expansion and methodological heterogeneity of MS approaches applied to epigenetics, a structured overview is needed to help researchers navigate analytical variability across sample preparation, instrumental platforms, and quantification strategies. By systematically mapping available workflows rather than evaluating biological outcomes, this review aims to provide a methodological frame of reference and identify areas where harmonization or further development is required.

We further narrow our scope to applications in human studies or in vitro models of human and animal origin.

## 2. Materials and Methods

This scoping review was conducted to map the existing research on the application of MS in the epigenetic field and to identify current knowledge gaps. The review followed the Preferred Reporting Items for Systematic Reviews and Meta-Analyses (PRISMA) guidelines [11] to ensure transparency and completeness in reporting. Although the protocol was not preregistered, it is available upon request from the corresponding author.

### 2.1. Search Strategy and Sources

A comprehensive bibliographic search was conducted using the three most popular electronic databases Web of Science, PubMed, and Scopus, selecting the most relevant studies published between 2015 and 2025 without any linguistic restriction. We sought to identify articles focused on the identification and quantification of epigenetic modifications using MS approaches. To this end, the following initial research questions were posed by the authors:

- Which epigenetic modifications are most frequently studied using MS?
- Which MS instruments are most employed?
- Which studies apply MS in clinical contexts or in human biological models?
- Have any new approaches or improvements been developed in sample preparation and data analysis specific to the application of MS to epigenetics?

### 2.2. Search Terms

**Figure 1:**
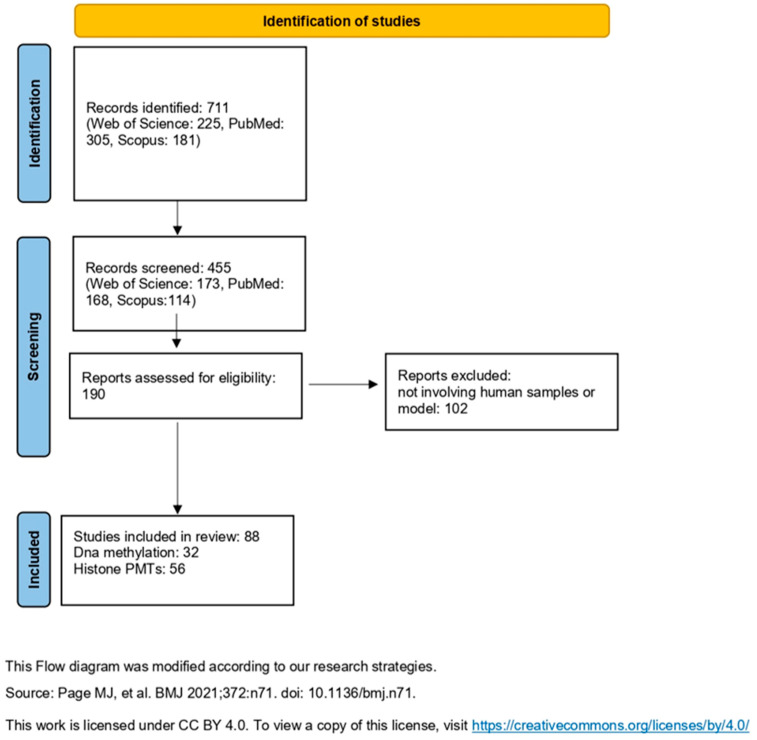
Overview of the workflow. Adapted from Page, M.J. et al. [12], licensed under CC BY 4.0. To view a copy of this license, visit https://creativecommons.org/licenses/by/4.0/ (accessed on 6 October 2025).

We focused our research solely on articles, by using the following strings in abstract title and keywords: “epigenetic modification” AND “quantification” AND “mass spectrometry”; “DNA methylation” AND “quantification” AND “mass spectrometry”; “histone modification” AND “quantification” AND “mass spectrometry”; “epigenetic modification” AND “mass spectrometry” and “treatment” AND “biological sample”; “DNA methylation” AND “mass spectrometry” and “treatment” AND “biological sample”, “histone modification” AND “mass spectrometry” and “treatment” AND “biological sample. Full-text articles were obtained online. Figure 1 illustrates the workflow adopted to identify, screen, and select studies in this review.

## 3. Results

The search results retrieved a total of 711 articles, of which 225 studies from Web of Science, 305 from PubMed, and 181 from Scopus. The authors selected articles based on the abstract and reviews following the questions written in Section 2.1 identifying 173 articles through Web of Science, 168 through PubMed, and 114 through Scopus. After thorough de-duplication, a consolidated set of 190 unique articles was obtained. From this final collection, only 88 articles specifically focused on applications in human samples or human models.

Results were summarized as follows: epigenetic modifications identified by MS, analytical method used to study DNA methylation and histone post-translation modifications (PMTs).

### 3.1. Epigenetic Modifications Identified by Mass Spectrometry

DNA methylation is a biochemical process where a cytosine residue is enzymatically methylated with a methyl group (–CH3) at the five carbon position [13,14]. This process predominantly occurs at the CpG sites (dinucleotide cytosine-phospho-guanine), which are present at high frequency in regions known as CpG islands (CGI). These regions are usually found in gene promoters, where gene expression is regulated through methylation [15,16]. Approximately 4% of cytosines appear in CpG context, and 60–80% of CpG cytosines are methylated depending on the cell type and physiologic or pathologic state [17]. DNA methylation is coordinated by a family of enzymes named DNA methyltransferases (DNMTs). It is a reversible chemical modification, and active demethylation processes are mediated by erasing DNA methylation mechanisms, mainly controlled by Ten-Eleven Translocation (TET) enzymes [18]. TET have been shown to catalyze the conversion of 5 methyl cytosine (5mC) to 5-hydroximethyl cytosine (5hmC) as well as into 5-formylcytosine (5fC) and 5-carboxylcytosine (5caC) [19]. DNA methylation has been shown to play an important role in the regulation of many essential biological processes, including retrotransposon silencing, genomic imprinting, X-chromosome inactivation, regulation of gene expression, and maintenance of epigenetic memory [20].

Acetylation, methylation, phosphorylation, and ubiquitylation are well-studied modifications that are known to significantly impact gene expression [3]. Aberrant histone acetylation and methylation in cancer alter the transcription of cytokines, chemokines, and transcription factors. This impacts the function and differentiation of T cells and tumor-associated macrophages (TAMs), key components of the tumor microenvironment (TME), leading to TME immunosuppression [21]. Numerous transcription factors, histone-modifying enzymes, and components of the transcriptional machinery interact with specific histone PTMs in a coordinated fashion, thereby influencing DNA function. Alterations in the regulation of histone variants and their post-translational modifications have been linked to several human diseases, including cancer. The number of PTMs that can be analyzed is several. Histones, H3 and H4 N-termini, in particular, are highly enriched in the number of possible modifications, both on the same residue and on neighboring residues [22].

Among the reviewed articles, 32 articles have been found to focus on DNA methylation, whereas 56 articles are focused on histone PMTs analyzed by MS.

### 3.2. DNA Methylation

Approximately 53% (17/32 studies) of the studies applied MS to human samples, including blood, plasma, urine, and tissues, while 40% focused on human cell lines. Among the remaining articles, one was focused on the analysis of both human samples and cell lines. The other was focused on a standard solution.

The most common instrument was the triple quadrupole (QqQ) mass spectrometer, employed in 69% (22/32) of the articles, including various QTRAP (Quadrupole Linear Ion Trap Mass Spectrometry) systems and dedicated triple-Q mass spectrometers. Other detection methods, such as Q-TOF/TOF (Quadrupole–Time of Flight/Time of Flight Mass Spectrometry), Orbitrap, ICP-MS (Inductively Coupled Plasma–Mass Spectrometry), MALDI (Matrix-Assisted Laser Desorption/Ionization–Time of Flight Mass Spectrometry) or DESI-TOF (Desorption Electrospray Ionization–Time of Flight Mass Spectrometry) were utilized in the remaining 31% of the studies.

In terms of chromatographic separation, reversed-phase C18 columns are used in 25% of the employed methods, reflecting their robust retention and compatibility with nucleoside analysis. Other types of columns such as hydrophilic interaction liquid chromatography (HILIC), mixed-mode or graphitised carbon columns and C8, were used less frequently. Mobile phase compositions are largely dominated by aqueous phases containing volatile buffers (e.g., 0.1% formic acid, ammonium acetate or ammonium formate), combined with organic phases such as methanol or acetonitrile.

Regarding sample pre-treatments, the majority of studies (19/32, 59%) adopt enzymatic hydrolysis of extracted DNA into nucleosides prior to MS analysis. These studies use multi-enzyme digestion protocols involving nuclease P1 (or S1 nuclease), phosphodiesterase and alkaline phosphatase. Five studies (16%) use acid hydrolysis with strong acids (e.g., formic acid or hydrochloric acid) at elevated temperatures, typically to shorten processing times, but with the potential risk of base degradation. Other studies apply alternative treatments, such as chemical derivatisation (e.g., benzoylation or dansylation) to improve chromatographic resolution or ionization efficiency, chemoenzymatic labeling of specific modifications (particularly 5hmC) or direct analysis of digested oligonucleotides rather than free nucleosides. These pre-analytical workflows directly influence the sensitivity, selectivity and susceptibility to artifacts of methylation analysis.

Quantification of methylated cytidine is essentially ever-present, 27/32 papers (84%) include 5mC or 5mdC among the monitored targets. Hydroxymethylcytosine is analyzed in approximately 20/32 studies (62%). Less frequently reported analytes include formylcytosine (5fC) (6/32 studies and carboxylcytosine (5caC) (6/32studies). Other modifications that are monitored include oxidized guanine bases (e.g., 8-oxodG), methylated purines (various N-methyl guanines) and rarer modifications such as N6-methyladenine. Collectively, these non-cytosine modifications appear in a minority of studies. Table 1 lists the results in order of publication year.

### 3.3. Histone PMTs

Among the 56 articles, 75% used cell lines (42/56), 7 studies analyzed human samples (12%), 2 human primary-derived monocytes, one PMBS, and one in standard solution. Among all the considered studies, 25 relied on cancer/tumor (46%). In particular, 19 were cell lines, 4 were human clinical samples, and 2 included both. This highlights a strong prevalence of cell line–based models compared to patient-derived material. The most frequently used lines were HeLa (31%, 13/42).

The bottom-up approach was the dominant sample preparation strategy by far. Acid extraction was the preferred method for histone isolation, with sulfuric acid being the most used one (59%, 33/56). The TCA (trichloroacetic acid) precipitation step is often employed.

Chemical derivatisation was dominated by propionylation (53%, 30/56), with propionic anhydride being the most used chemical. Acetylation is used only in 9% of studies (5/56). On the other hand, trypsin is largely used for the digestion step (86%, 48/56). The final crucial step of desalting was predominantly performed using C18 StageTips to remove impurities that could interfere with MS analysis.

The gold standard for peptide separation was liquid chromatography (LC), with C18 reversed-phase columns dominating (77%, 43/56). Mobile phases typically consisted of a binary mixture of formic acid in water (solvent A) and formic acid in acetonitrile (solvent B), a well-established system for compatibility with MS.

The MS platforms most frequently used were from the Orbitrap family (Q-Exactive/Fusion/Lumos; 66% 37/56), favored for their superior mass accuracy and high resolving power, followed by triple quadrupoles/QTRAP (16%, 9/36), TripleTOF/TOF (7%, 4/56). Imaging MS was used in 7% of the studies (4/56) with MALDI-TOF instruments.

The dominant data acquisition method was data-dependent acquisition (DDA) (52%, 29/56), a discovery-based approach that randomly selects the most abundant peptides for fragmentation. An increasing number of studies employed data-independent acquisition (DIA) or SWATH strategies (20% and 5%, respectively), which provide more comprehensive and reproducible peptide maps. Targeted multiple reaction monitoring (MRM) (18%, 10/56) and parallel reaction monitoring (PRM) (5%, 3/56) were used for focused quantification. Table 2 resumes the results in order of publication years.

## 4. Discussion

### 4.1. DNA Methylation

The collected data clearly show a community preference for targeted, quantitative LC–MS/MS on triple-quadrupole platforms for DNA methylation and nucleoside quantification. This choice reflects the need for high sensitivity and the routine use of multiple reaction monitoring (MRM/SRM) methods for quantifying low-abundance modified nucleosides in complex biological matrices.

However, the sample pre-treatment methods show significant variability, with enzymatic hydrolysis being the most frequently employed approach. Some studies adopt hybrid approaches, combining chemical and enzymatic steps to balance speed and fidelity, though optimization is critical to avoid inconsistent recoveries.

A key challenge that has been identified is the need for highly effective purification and pre-concentration steps. Several studies have mentioned the use of solid-phase extraction (SPE) or specific precipitation methods to address matrix effects and improve recovery. These patterns suggest that the field is moving towards two complementary strategies: enzymatic digestion and targeted LC–QqQ quantitation for robust quantification, and labeling/derivatisation or high-resolution mass spectrometry (HR-MS) approaches for extreme sensitivity or the broader discovery of low-abundance species. Chemical modifications are increasingly being used to improve the efficiency and sensitivity of ionization. Examples include labeling 5hmdC with beta-glucosyltransferase, derivatisation with 4-(dimethylamino)benzoic anhydride or chemical labeling for the targeted analysis of oxidized nucleosides. Although HR-MS offers powerful structural capabilities, it may exhibit a reduced dynamic range and often requires more complex data processing, ultimately limiting throughput. In parallel, some studies have explored MALDI-TOF [28,44] and LAMP-TOF [51] for site-specific methylation analysis, providing rapid screening options, albeit with lower sensitivity than LC-MS/MS.

An additional consideration emerges from the observed trends in chromatography and the mobile phase. Although the prevalence of C18 reversed-phase columns (around 74%) demonstrates their versatility in nucleoside analysis, they can struggle to retain highly polar analytes such as 5hmC or 5fC. This could justify the use of HILIC phases (approximately 19%) or graphitized carbon supports (approximately 7%) in targeted workflows.

Nevertheless, the review highlights several critical limitations. A major issue is theinconsistent reporting: while most studies provide some validation parameters, many fail to present a comprehensive validation set, including matrix effects, recovery across the analytical range, and stability assessments. The absence of such information complicates cross-study comparisons and undermines the feasibility of meaningful meta-analyses. This gap poses a significant challenge for the scientific community as it limits the ability to evaluate data quality and reproduce results across laboratories. Ultimately, it compromises the robustness and reliability of the findings.

Secondly, methodological choices inherently involve trade-offs. Enzymatic hydrolysis protocols are generally preferred due to their ability to reduce the risk of artefactual degradation. Indeed, enzymatic digestion is gentler and better preserves oxidized cytosine derivatives, improving accuracy for low-abundance modifications. The main drawback of enzymatic digestion is the increased complexity and duration of multi-step protocols, which may introduce variability if enzyme activity or incubation conditions are not rigorously controlled. In contrast, strong-acid hydrolysis is a simpler and faster approach, but the harsh conditions may introduce artificial base modifications. Rapid chemical hydrolysis is convenient, but it may introduce bias in the quantification of labile marks. It typically involves high-temperature, FA treatment and provides rapid and straightforward DNA cleavage, making it suitable for high-throughput applications. However, this approach can induce partial degradation of labile modifications, particularly 5hmC and 5fC, which could lead to an underestimation of these marks.

The impact of matrix effects is a recurring issue, as highlighted in several articles which emphasize the need for specific purification and pre-concentration steps, such as SPE. Endogenous compounds present in complex biological matrices (e.g., serum, urine or tissue lysates) can interfere with the MS signal, resulting in inaccurate quantification. While some studies address this with specific protocols, there is no consistent, robust approach yet to mitigate these effects. Pre-analytical heterogeneity (e.g., different DNA extraction kits, inconsistent use of internal standards (IS), and varied cleanup procedures) introduces potential biases: reported recoveries span a wide range, and not all studies correct for recovery or matrix suppression using isotopically labeled IS. The lack of a single, universally adopted protocol contributes to variability between methods. The diversity of sample preparation methods, combined with the lack of comprehensive validation reporting, highlights the urgent need to develop and adopt harmonized protocols to ensure the quality, comparability, and reliability of DNA methylation data in research and clinical settings. Moreover, studies frequently prioritize technical precision over addressing potential pre-analytical sources of error, such as DNA degradation during storage or extraction. This can have a disproportionate impact on oxidized derivatives. Furthermore, while triple-quadrupole MS remains the gold standard for targeted quantification, the relatively limited application of HR-MS contrasts with its widespread use. HR-MS would allow for the more confident identification of unexpected or novel nucleoside modifications and better resolution of isobaric interferences. Meanwhile, MALDI-and TOF-based approaches, although adopted by only a minority of studies, offer certain advantages, such as higher throughput and positional information (e.g., EpiTyper MALDI assays). However, these methods generally have lower quantitative sensitivity than QqQ platforms. MALDI-TOF in particular suffers from a restricted dynamic range and limited accuracy in measuring methylation levels, despite its promise.

Finally, many reports have limited sample sizes and clinical validation (small cohorts and single-center measurements).

However, to advance clinical translation and enable reliable cross-study synthesis, future work should emphasize full method validation (including matrix effect assessment and stability), wider adoption of isotopically labeled standards, and coordinated inter-laboratory standardization, while preserving the complementary role of high-resolution and derivatisation-based methods for discovery and applications requiring extreme sensitivity. This convergence would ultimately improve the reproducibility of methylation biomarker studies and accelerate the transition from analytical development to clinically actionable assays.

### 4.2. Histone PMTs

A critical evaluation of these findings shows that, despite being well-established, the field of histone proteomics faces significant challenges that prevent it from reaching its full potential. The data show a strong trend towards in vitro cell line models (48 articles), with only a small proportion of studies (8 articles) utilizing direct human samples. This methodological preference has important implications for the applicability of research findings. While these models offer advantages such as reproducibility and experimental control, their altered and simplified biology may not accurately reflect the complex PTM dynamics observed in living human organisms or tissues. The limited use of human samples, predominantly blood-derived cells, highlights the logistical challenges of obtaining and processing more complex clinical specimens. The discrepancy between in vitro and clinical models remains a critical bottleneck in the field.

While technically sound, the heavy reliance on the bottom-up approach is a major impediment to deciphering the histone code. Although this method simplifies the analytical process by breaking down histones into smaller peptides, it has a significant and frequently overlooked drawback: it destroys information about PTM combinatorial patterns on a single histone molecule. The loss of this ‘PTM crosstalk’ is a fundamental limitation that prevents a complete understanding of the complex biological code encoded by multiple PTMs on a single histone molecule. Identifying individual modifications is not enough; the biological meaning resides in their specific combinations. The fact that only a handful of studies have explored middle-down or top-down proteomics highlights a methodological gap that the field must address to gain a more comprehensive understanding of histone PTM dynamics.

Concerning the sample pre-treatment, the subsequent core procedure is proteolytic digestion, primarily performed using trypsin to achieve robust peptide coverage for bottom-up analyses. However, trypsin can cleave after methylated or acetylated lysines, which makes identifying PTM sites challenging. To address this issue, many protocols incorporate propionylation (either before or after digestion) to seal off the free N-termini of lysine and prevent non-specific enzymatic cleavage. This makes it easier to localize the marks of methylation and acetylation with confidence. However, the strong reliance on propionylation improves chromatographic behavior and tryptic specificity; incomplete propionylation, variability in derivatisation efficiency between samples, and side reactions on certain acyl marks can distort stoichiometry. Alternative approaches, such as acylation using acetic or deuterated acetic anhydride, and alkylation using dithiothreitol or iodoacetamide, offer partial solutions, but they can introduce artifacts or fail to capture transient modifications. Furthermore, enzymes such as GluC are used in middle-down approaches to produce longer peptides, which are beneficial for analyzing PTMs that are far apart on the histone tail. This preserves combinatorial information that is often lost in bottom-up methods.

Rigorous inclusion of internal heavy standards is still not systematic. On the other hand, although acid extraction is fast and efficient, it risks disturbing labile PTMs (e.g., some acylations and phospho-marks), and when combined with extensive TCA precipitation, it can result in losses or introducing matrix components that complicate ionization.

The most prevalent bottom-up approaches afford high site-specific resolution, but obscure combinatorial PTM patterns. Conversely, middle-down and top-down analyses preserve such patterns, but require greater sample input, complex workflows, and advanced instrumentation, which limits their routine adoption.

In the field of MS, targeted methods such as MRM and PRM improve quantitative reliability but limit global discovery. In contrast, DDA and DIA approaches enable broader mapping but sacrifice sensitivity to low-abundance marks. Orbitrap-centric DDA offers high mass accuracy and extensive coverage, but is affected by stochastic precursor sampling and co-isolation interference. DIA/SWATH is used for a significant proportion of applications, yet remains underutilized despite its advantages in reproducible quantification. This is probably due to the limited availability of histone-tail-specific spectral libraries and turnkey scoring workflows. The shift from DDA to DIA is particularly encouraging. DIA enables comprehensive, systematic, and quantitative analysis by acquiring all fragment ion spectra, thus overcoming the stochasticity of DDA and providing a more complete dataset. Developing advanced software and bioinformatic pipelines is also crucial for interpreting the immense complexity of the data generated by these high-throughput techniques.

Overall, these statistics demonstrate the ongoing challenge of balancing methodological compromises. High-throughput and sensitive quantification often come at the expense of combinatorial context and reproducibility. Conversely, approaches that capture PTM complexity tend to be less scalable and more demanding in terms of instrumentation. However, the technological landscape offers some promising solutions. Adopting new-generation instruments such as modern Orbitrap and Q-TOF instrumentation represents a significant advance. These hybrid instruments combine the speed of a linear ion trap with the high resolution of an Orbitrap, enabling faster and more precise analysis. Overall, the weak link is reproducibility: extraction protocols, derivatisation efficiency, choice of acquisition mode, and variable clean-up steps all create avoidable variance between studies.

Looking to the future, several areas are essential for the field to progress. Firstly, there is an urgent need for standardized protocols to ensure inter-laboratory reproducibility and facilitate data sharing. The development of community reference materials and internal standards is crucial for benchmarking acid versus extraction, codifying propionylation protocols (including checks for over-derivatisation), and reporting QC metrics such as digestion efficiency.

Secondly, research should focus on developing and implementing methodologies that preserve PTM combinatorial information, such as combined top-down/middle-down approaches. DIA should be adopted more broadly alongside openly shared, histone-focused libraries, and DIA/PRM should be paired with rigorous interference evaluation. Finally, software pipelines should be consolidated (with open formats, versioned parameters, unit-tested quantification and ready-to-reuse Skyline/EpiProfile-style templates).

### 4.3. General Observations Across MS-Based Epigenetic Workflows

In addition to the heterogeneity observed across analytical workflows, several studies highlight the importance of addressing the stability of epigenetic marks during sample preparation and storage. A subset of the reviewed articles includes oxidized nucleobases such as 8-oxo-dG, 5-formyl-dC or 5-carboxyl-dC among their analytical targets, underscoring their relevance not only as markers of oxidative stress but also as potential artifacts introduced during DNA extraction, storage, and hydrolysis (e.g., references [25,38,47] in our review). This aspect has also been highlighted in the broader literature, where several authors note that improper sample handling can lead to spontaneous oxidation of guanine-or cytosine-derived species. For example, substantial discrepancies in reported endogenous levels of cellular 8-oxodG are largely attributed to artefactual oxidation of dG occurring during DNA isolation and processing [110]. Future methodological developments should therefore prioritize standardized protocols aimed at minimizing artefactual oxidation, including: (i) the systematic use of antioxidants (e.g desferrioxamine (DFAM) or butylated hydroxytoluene (BHT) [111]) during extraction; (ii) controlled-temperature workflows for hydrolysis and enzymatic digestion [112]; (iii) immediate stabilization and aliquoting to reduce freeze–thaw cycles [112]; and (iv) validated storage conditions for long-term biobanking. Harmonizing such practices across laboratories would significantly improve reproducibility, especially in studies quantifying low-abundance modified nucleosides. Integrating these stabilization strategies into MS-based epigenetic pipelines represents a key future direction for the field.

Although the primary focus of this scoping review is the analytical variability of MS-based approaches for epigenetic analysis, it is worth noting that several articles in the broader literature highlight additional practical aspects influencing the adoption of these methodologies. In particular, some authors underline that access to MS platforms, especially in laboratories without established analytical facilities, and the need for operators with specific training in sample processing and data interpretation may represent relevant barriers to widespread implementation [113,114,115,116,117].

## 5. Conclusions

Overall, while the field benefits from advanced MS technologies and diverse analytical targets, future work should prioritize methodological standardization, comprehensive validation reporting, and integrated multi-omics approaches to enhance biological insight and clinical translational potential.

The ultimate challenge will be to integrate histone proteomics and DNA methylation data with other ‘omics’ platforms (e.g., transcriptomics and genomics) to reveal the entire network of epigenetic regulation. Histone PTM and DNA methylation research will only evolve from a characterisation tool to a means of understanding the molecular mechanisms of health and disease by combining robust methods, state-of-the-art instrumentation, and sophisticated bioinformatics.

## Figures and Tables

**Table 1:** Analyte quantification, sample pre-treatment, validation parameters and instrument used in the reviewed papers.

Ref, Year	MS Quantification	Samples	Sample Pre-Treatment	Validation Parameters	Instruments
[23], 2015	5mdC	A549 human lung adenocarcinoma epithelial cell line and A2780 human ovarian carcinoma cell line	Extraction: PureLink Genomic DNA Mini Kit (Invitrogen, Carlsbad, CA, USA). Enzymatic Digestion: Nuclease S1 at 37 °C Purification: membrane ultracentrifugation with a Centricon YM-10 filter device.	%RSDs < 4%. LOD: 40–41 ng mL^−1^ for 5mdCMP and dCMP (measured element (31P) with same stoichiometry (1:1) in all nucleotide monophosphate)	System: LC-2 double-focusing magnetic sector field ICP-MS Column: A Mono-Q™ column
[24], 2015	5hmdC, 5mdC, dC, dG	Metastatic melanoma cell line WM266–4 and leukemia cell line KG1	DNA Extraction: Blood & Cell Culture DNA Mini Kit (Qiagen, France). DNA Hydrolysis: Denaturation: at 100 °C for 3 min Enzymatic Digestion: Step 1: Nuclease P1 at 45 °C for 2 h. Step 2: Venom phosphodiesterase I at 37 °C for 2 h. Step 3: Alkaline phosphatase 1 h at 37 °C.	Not Reported	System: LC-Q TRAP Column: Polar-RP 80A. Eluent A: 0.1% FA in H_2_O Eluent B: 0.1% FA in MeOH
[25], 2015	5mC, 5hmC, 5fC, 5caC	human urine	SPE: HLB cartridge; Elution: First Elution: 1:9 MeOH/H_2_O solution. Second Elution: 3:7 MeOH/H_2_O solution Drying & Reconstitution: H_2_O	Recovery %: 101.3 ± 4.1% for 10 nM 5hmC, 103.5 ± 2.4% for 20 nM 5hmC, 70.2 ± 0.9% for 10 nM 5mC, and 89.9 ± 0.6% for 20 nM 5mC. Inter-day precision (RSD%): from 2.9% to 10.6%, and the intra-day: from 1.4% to 7.7%. LODs: 25 amol for 5mC and 250 amol for 5hmC; LOQs: 75 amol for 5mC and 760 amol for 5hmC.	System: LC-QqQ Column: NUCLEOSHELL RP 18 Eluent A: 2.0 mM NH_4_HCO_3_ in H_2_O, pH 8.5 Eluent B: 100% MeOH
[26], 2015	5-mC, 5-hmC	human blood.	DNA Extraction: E.Z.N.A. Blood DNA Kit (Omega Bio-Tek Inc., Norcross, GA). Enzymatic Digestion: Step 1: S1 nuclease at 37 °C for 4 h. Step 2: Alkaline phosphatase and venom phosphodiesterase 2 h at 37 °C. Purification: phenol/chloroform extraction, followed by two extractions with chloroform Reconstitution: ACN/H_2_O.	LODs for 5-mC and 5-hmC were 0.04 and 0.13 fmol RSDs% and relative errors <11.2% and 14.0%, respectively.	System: on-line trapping/cHILIC/micrOTOF-Q Columns: on-line trapping column: 0.5 cm poly(MAA-co-EGDMA) monolithic capillary. Analytical column: 30 cm hydrophilic organic-silica hybrid monolith. Eluent A: 0.01% FA in H_2_O. Eluent B: 0.01% FA in ACN
[27], 2015	Cyt, 5mdC	Normal breast cell line MCF10A, breast cancer cell line MCF7.	Hydrolysis: 1 mL of FA in a muffle furnace at 140 °C for 1.5 h. Drying and Reconstitution: 1 mL of MeOH	Not reported	System: LC-Orbitrap
[28], 2015	DNA methylation patterns in the GSTP1 promoter	Colon cancer and normal control tissues	DNA Extraction: Wizard Genomic DNA Purification kit (Qiagen, Germany). Sample Preparation: GOOD assay Matrix Preparation: matrix solution of α-cyano-4-hydroxycinnamic acid methyl ester in acetone Sample and Matrix Spotting: spotted onto the target plate using a robot. A small volume of the DNA sample pipetted onto the top of the dried matrix.	Not reported	System: MALDI-TOF Acceleration potential: ±18 kV, and ion extraction is delayed by 200 ns.
[29], 2016	5‘-mdC 5‘-hmdC	human liver cancer tissue	DNA Extraction and Quantification: Takara MiniBEST universal Genomic DNA Extraction Kit (Takara Bio, Dalian, China).	LODs: 5 amol and 10 amol for 5′-mdC 5′-hmdC; Intra-and inter-day RSDs% < 6.2% and 7.9%. Intra-day RSD% for the peak area: 7.4%; Inter-day RSD for the peak area: 8.4%	System: CE-Orbitrap Capillary: A 100 cm long bare fused-silica capillary with a 30 µm internal diameter Capillary Preparation: flushed with MeOH, H_2_O, 0.1 M NaOH, 0.1 M HCl, and finally with the background electrolyte (BGE), which is 10% acetic acid (pH 2.2).
[30], 2017	5mC, 5hmC, 5fC, 5caC	Global human breast cancer and tumor-adjacent normal tissue genomes.	Extraction: extraction kit (Puhe Bio-Tech Co. Ltd., Wuxi, China) Enzymatic Digestion: Step 1: incubated with Cryonase Cold-active Nuclease at 40 °C for 1 h. Step 2: Alkaline phosphatase and phosphodiesterase I at 37 °C for an additional 4 h. Purification: NaCl and absolute ethanol Derivatization: 4-(dimethylamino) benzoic anhydride.	LODs and LOQ of 5-mC, 5-hmC, 5-foC, and 5-caC: 1.2~2.5 fmol and 3.7~7.6 fmol, Intra-and inter-batch RSDs: 3.1~6.3%, 3.4~5.1% for 5-mC; 0.9~5.5%, 1.6~5.5% for 5-hmC; 0.8~12.9%, 2.3~9.9% for 5-foC; 5.1~9.1%, 3.8~7.2% for 5-caC	System: LC-Q TOF Column: Eclipse Plus C18 column and UPLC SB C18 column.
[31], 2017	5-fodC, 5 forC, 5-fodU, 5-forU, 5 forCm, 5-forUm,	Human embryonic kidney cells (293T), human breast cancer cells (MCF-7) Tissue samples from thyroid carcinoma patients	Extraction: E.Z.N.A. HP kits (Omega Bio-Tek Inc., Norcross, GA, USA) Enzymatic Digestion: Step 1: at 95 °C, then incubated with S1 nuclease at 37 °C for 2 h. Step 2: Alkaline phosphatase and venom phosphodiesterase I 2 h at 37 °C. Purification: chloroform extraction to remove proteins; graphitized carbon black SPE cartridge Labeling: The purified and dried sample is then chemically labeled using specific reagents (GirP, GirT, and 4-APC) for targeted analysis.	LODs and LOQ: 0.03 and 0.10 fmol for 5-fodC,0.04 and 0.12fmol for5 for C,0.05 and 0.17fmol for5-fodU and 0.05 and 0.18 fmol for5-forU. Intra-and inter-day RSDs for four GirP-labeled < 13.2% and 14.3%, respectively;	System: LC-QqQ SPME Column: A poly(MAA-co-EGDMA) monolith as an in-tube SPME column for sample loading and washing. Analytical Column: Shim-pack ODS column Eluent A: 0.1% FA in H_2_O Eluent B: 0.1% FA in MeOH
[32], 2018	dC, dT, dA, dG, 5mdC, 5hmdC (converted in N3-5gmdC)	blood samples from healthy individuals and leukemia patients,	Extraction: DNA Tissue kit (5 Prime, Hilden, Germany). Protein Removal: proteinase K overnight at 55 °C. Filtration: Amicon Ultra 0.5 mL 10K columns. Enzymatic Hydrolysis: Step 1: DNA samples are heat-denatured at 98 °C for 5 min. Nuclease S1 at 37 °C for 3 h. Step 2: Antarctic phosphatase and phosphodiesterase at 37 °C Inactivation: at 80 °C for 10 min in the presence of EDTA. Labeling Strategy: β-GT enzyme: convert 5hmC into N3-5gmC	Not reported	System: LC-QqQ Column: Xselect HSS T3 column Eluent A: 0.1% FA in H_2_O Separate solvent containing 0.1% FA.
[33], 2018	dC, 5mdC, 5hmdC	375 melanoma, A2058 melanoma, HepG2 hepatocarcinoma, HeLa cervix carcinoma, MES-SA uterine sarcoma, H1650 bronchoalveolar carcinoma, HTR8 placenta, BeWo choriocarcinoma, HL60 promyeloblast, and K562 lymphoblast cell lines	DNA Chemical Hydrolysis: 100% FA heated at 130 °C for 90 min	Not Reported	System: LC-Q TRAP Column: Agilent RX-Sil Eluent A: H_2_O + 0.1% FA. Eluent B: ACN + 0.1% FA.
[34], 2018	5hmdC	ladder cancer (T24) cells	BenzoSAC bioreactor Digestion: Benzonase, snake venom phosphodiesterase (SVP), and alkaline phosphatase (ALP).	LOD:0.2 nM LOQ: 0.5 nM Recovery%: 85.4 ± 5.9%, 92.5 ± 4.8% and 102.2 ± 3.2% for 2, 10, and 100 nM of 5hmC, respectively. intraday RSD%:4% Inter-day RSD%:6.6%	System: LC-QqQ Column: Zorbax Eclipse Plus C18: Eluent: 5% MeOH and 95% 2 mM ammonium bicarbonate solution.
[35], 2018	5mdC,5-hmdC	Human urine of CRC patients CRC	SPE: MCX cartridge, elution with CH3CN/H_2_O/NH4OH (90:10:5, *v*/*v*/*v*).	LODs for 5-mdC, 5 hmdC, 5-mrC and 5-hmrC being 0.025, 0.025, 0.025 and 0.050fmol, respectively. RSD%: 0.20% to 2.63%. Accuracy%: 98.19–109.54%	System: LC-Q TRAP Column: BEH HILIC Eluent A: ACN containing FA, ammonium formate, and malic acid. Eluent B: H_2_O containing FA and ammonium formate.
[36], 2019	dc, 5mdc	Human blood	DNA Sample Preparation Extraction: QIAamp DNA Mini Kit (Qiagen, Germantown, MD, USA). Enzymatic Digestion: Step 1: Nuclease P1 at 65 °C for 10 min Step 2: Alkaline phosphatase at 37 °C for 1 h	Recoveries%: 31.0% for 2dC, 32.3% for 2dC, 21.9% for 5mdC. LLOQ of 50 ng/mL and 5 ng/mL for 2dC and 5mdC, respectively. Bias (%) from −5.2% to 9% CV% from 0.9% to 12.6%	System: LC-Q TRAP Column: H_2_Os Nova-Pak Silica Eluent A: 5 mM ammonium acetate in H_2_O Eluent B: 5 mM ammonium acetate in a H_2_O-ACN mixture
[37], 2019	Guo, uro, cyt, 5-mdU, dU, dC, dA, mdC, hmdC, dT, dG, 5-Aza-dC	Human pancreas carcinoma cell lines (MIAPaCa-2, AsPC-1, and BxPC-3),human colorectal carcinoma cell lines (HT29, Caco-2, and HCT116), human breast cancer cell lines (SKBR, CF-7, MDAMB231, MDAMB468, and T47D	DNA Hydrolysis Protocol: Denaturation: at 95 °C for 3 min, Enzymatic Digestion: DNA Degradase Plus, nuclease P, PDE, and ALP and incubated at 37 °C for 2 h Inactivation: at 70 °C for 20 min.	intra-day CV% < 3% inter-day CV%< 5%.	System: LC-QqQ Columns: Acquity UPLC BEH Phenyl Column Eluent A: 0.1% ammonium bicarbonate in H_2_O Eluent B: 0.1% ammonium bicarbonate in 90% MeOH
[7], 2019	1. 5mdC, 5hmdC, 5fdC, 5cadC 8oxodG 2. 4mdC and 6mdA	NGN cells66 HEK293T cells mES wild-type cell line J1	gDNA Isolation: Zymo-Spin IIC-XL spin column (Zymo Research Corp, Irvine, California, USA). DNA Digestion: two separate enzyme mixes: Mastermix 1: Nuclease S1 and ZnSO_4_. Mastermix 2: s snake venom phosphodiesterase I and EDTA.	LLOQ: from 6.5 × 10^−4^ to 0.1 pmol; ULOQ: from 0.47 to 228.6 pmol	System: LC-QqQ Method 1: Column: C8 Method 2: Column: C18 Mobile Phases: Solvent of a H_2_O/ACN
[38], 2019	5mC, 1MeG, 6MeG, 7MeG, 9MeG 2EtdG, 1MeA, 3MeA, and 9MeA 5hmC and 5mdC MeA, 9EtA and 9EtG	Human kidney cell line 293 T and the human liver cell line L02 exposed to 3 genotoxic reagents: N-methyl-N-nitrosourea (MNU), methyl methanesulfonate (MMS) and 4-(methylnitrosamino)1-(3-pyridyl)-1-butanone (NNK).	DNA Extraction: QIAamp DNA mini kit (Qiagen, Germantown, MD, USA). Hydrolysis: 1N HCl and heated at 85 °C for 1 h. Enzymatic Digestion: Step 1: Nuclease P1 and incubated at 37 °C for 2 h. Step 2: Alkaline phosphatase 2 h at 37 °C.	Accuracy: 82.1–115% Intra-day RSD%: <14% Inter-day RSD%: <15% Recoveries% of two pretreatment methods: 50.5% to 126%, ME%: NO LLOQs:0.25 ng/mL for 5hmC; 0.1 ng/mL for 1MeG, 7MeG, 9MeG and 2EtdG; and 0.05 ng/mL for the other analytes. LLODs: 50 pg/mL for 5hmC; 10 pg/mL for 1MeG, 7MeG, 9MeG and 2EtdG; and 5 pg/mL for the other analytes	System: LC-QqQ Column: H_2_Os ACQUITY UPLC BEH Amide Eluent A: H_2_O with 0.1% FA and 10 mM ammonium acetate. Eluent B: ACN with 0.1% FA.
[39], 2019	Cyt, urd, ino, 5-methyl-CMP, cyt monophosphate 5mdC	Serum samples	Protein Precipitation and Extraction: pre-chilled MeOH/chloroform mixture at −20 °C. Drying and Reconstitution: in mobile phase.	Inter-day RSD%: from 0.38% to 10.42% Intra-day RSD%: from 2.04% to 13.26% Recovery%: from: 81.55% to 115.69% LLOQ: from 0.02 to 195.30 ng/mL	System: LC-QqQ Column: H_2_Os XBridge Amide column Eluent A: H_2_O containing acetic acid, ammonium acetate, and succinic acid. Eluent B: ACN.
[40], 2019	5mC, 5hmC, 5fC, 5caC.	Tet1 overexpressed 293T cells	One-Step Hydrolysis: combination of enzymes: PDE1 & ALP Benzonase, PDE1 & ALP DNase 1, PDE1 & ALP Nucleoside Digestion Mix (from NEB) DNA Degradase Plus (from Zymo) Two-Step Hydrolysis: Step 1: P1 nuclease, PDE1, and ALP are incubated overnight. Step 2: The same enzyme mix is incubated for an additional 3 h.	Not reported	System: LC-QqQ Column: ZORBAX Eclipse Plus C18 Eluent A: 10 mM ammonium acetate (pH 6.0) Eluent B: MeOH
[41], 2020	Cyt, 5mC, 5hmC	Tissue samples from patients with PitNET were collected	DNA Extraction: QIAamp Fast DNA Tissue Kit (Qiagen, Germantown, MD, USA). and from blood samples QIAamp DNA Mini Kit (Qiagen, Germantown, MD, USA).. Hydrolysis: 0.2 mL of 98% FA and hydrolyzed by heating at 140 °C for 90 min.	Not reported	System: LC-Q TRAP Column: BEH HILIC column. Eluent A: H_2_O with 2.5 mM ammonium formate. Eluent B: ACN with 0.05% FA.
[42], 2020	dC, 5mC, 5hmC, and labeled 5hmC (oxidation to 5fC)	human plasma	cfDNA Extraction: QIAamp circulating nucleic acid kit (Qiagen, Germantown, MD, USA). cfDNA Hydrolysis: nucleoside digestion mix	5hmC level in the cfDNA sample (less than 5 ng). to 12.5 fmol Without labeling, LOD of 5hmC: 2.5 fmol After labeling: LOD14 amol intra-day: from 0.74% to 7.4%. Inter-day: from 7.2% to 11%	System: LC-QqQ Column: A Zorbax Eclipse Plus C18 Eluent A: H_2_O with 0.0085% FA (FA) Eluent B: MeOH with 0.0085% FA (FA)
[43], 2021	5mC, 5hmC	Human cell lines: HL60 (CCL-240) and K562 (CCL-243)	Hydrolysis: FA Incubation: 140 °C for 90 min. Drying: evaporated under a stream of nitrogen. Reconstitution: 50:50 ACN-H_2_O solution containing 0.1% FA	Accuracy and precision: range of 0.005–0.5% for 5hmC and 1–15% for 5mC. LOQ for 5hmC 1.43 fmol and 7.14 fmol for 5mC	System: LC-Q TRAP Column: Zorbax Rx-SIL Eluent A: H_2_O with 0.1% FA. Eluent B: ACN with 0.1% FA.
[44], 2022	Methylation of sixth intron of RPTOR harboring six CpG sites	Peripheral blood samples BC cases and healthy controls	gDNA Extraction: Genomic DNA Extraction Kit (Zymo Research, Orange County, CA, USA) Bisulfite Conversion: EZ-96 DNA Methylation Gold Kit (Zymo Research, Orange County, CA, USA). PCR & Purification	Not reported	System: MALDI-TOF
[45], 2022	5-mC, 5-hmdC, 5-cadC, 5-fdC, 5-hmdU	whole blood specimens of SSc and healty patients	DNA Extraction: High Pure PCR Template Kit (Roche, Germany). Enzymatic Digestion: Step 1: The DNA is mixed with Nuclease P1 and incubated at 37 °C for 1 h. Step 2: Alkaline phosphatase 37 °C for 1 h. Purification: 10 kDa cut-off membrane	Not reported	System: LC-QqQ Column: X-select CSH C18
[46], 2022	5mC, methylated cytosine	human serum sample	Enzyme: hOGG1, bisulfite conversion step.	LLOD: 84 pM, and a 0.1% methylation. Recovery%: between 96.7% and 105%, RSD%: 3.0–3.5%,	System: ICP-MS Label: lanthanide (169-thulium) for DNA methylation analysis. Nebulizer Gas Flow: 0.93 L/min Auxiliary Gas Flow: 1.2 L/min Plasma Gas Flow: 18 L/min ICP RF Power: 1300 W Isotope Monitored: 169-Tm
[47], 2022	8-Oxo-dG, εdA, N6-Me-dA, N2-Et-dG, O6-5-Cl-dC, 5-m-dC, 5-hm-dC	human embryonic lung fibroblast (HELF) cells	Nuclear Extraction: commercial kit and proteinase K at 55 °C for 2 h DNA Precipitation: sodium acetate, pre-cooled absolute ethanol Enzymatic Digestion: deoxyribonuclease at 37 °C for 2 h. Phosphodiesterase and alkaline phosphatase overnight. Purification: chloroform extraction; evaporated and redissolved in ultrapure H_2_O	LOD: 0.02pg (on-column), RSD% intra-day and interday precisions: 0.5–8.1 and 0.9–11.5% Accuracy: 85.7–113.0% ME%: 94.5%–108.5% Recovery%:106.7–113.7%	System: LC-QqQ Column: Thermo Hypurity advance Eluent: 5 mM ammonium acetate (pH 7.3) and ACN 95:5 (*v*/*v*).
[48], 2023	dC, dA, dG, dT 5mC, 6 mA	Cells lines HEK 293 and HEK 293T	Enzymatic Digestion: Benzonase and SVP, and incubated at 37 °C for 8 h. CIP is then added for an additional 1 h of incubation. Purification: ultrafiltration tube	Not reported	System: LC-QqQ Column: Zorbax Eclipse Plus C18 Eluent A: 2.0 mM NH_4_HCO_3_ in H_2_O Eluent B: 100% MeOH
[49], 2023	dG, O6-Me-dG	Pure solutions	Matrix Preparation: Graphene in ethanol/TFA DHPT in MeOH/TFA 3-HPA in H_2_O DHB in MeOH/H_2_O/TFA CHCA in MeOH/H_2_O/TFA DESI Sample Preparation: Sample spotted onto a specialized Aquarray DMA Slides.	Not reported	System: Synapt G2-Si DESI-Q-IM-TOF
[50], 2023	m6dA, 5-mdC, 5-hmdC, dA, dG, dC, T, Ado, Guo, Cyt, Urd, 5-mdC, 5-hmdC	human tumor and healty tissues	DNA Extraction: QIAamp DNA Mini Kit (Qiagen, Germantown, MD, USA).. Enzymatic Digestion: Step 1: NP1, PDE2, and EHNA at 37 °C for 48 h. EHNA Step 2: Quick CIP at 37 °C for an additional 2 h. Purification: chloroform extraction.	LODs: 0.005 to 0.25 nM; LOQs: 0.05 to 1 nM; accuracy and precision: intraday: RSD%: 0.16% to 3.54%; accuracy: 94.79% to 104.84%. Interday: RSD%: 0.09% to 2.65%; accuracy: 91.63 to 104.78	System: LC-Q TRAP Column: H_2_Os BEH Amide Eluent A: H_2_O with 0.2% FA, 10 mM ammonium formate, and 0.05 mM malic acid. Eluent B: ACN with 0.2% FA, 2 mM ammonium formate, and 0.05 mM malic acid.
[51], 2025	5mC, cyt	Plasma of CRC and healthy patients.	Extraction: VAHTS™ Serum Plasm Circulating DNA Kit (Vazyme, China) cfDNA Extraction: Ligation and Bisulfite Treatment: cytosine to uracils Amplification:T7 RNA polymerase Linear Amplification: PCR Amplification Incorporation of a specific dideoxynucleotide for the methylation status: ddATP: unmethylated dU). ddGTP:methylated (5mC)	Not reported	LAMP-TOF
[52], 2025	5mC, 2omdC	MDA-MB-231 breast cancer cells, HEK293 cells	DNA Extraction: QIAwave DNA Blood & Tissue Kit (Qiagen, Pittsburgh, PA, USA). Cell lysis with proteinase K and Buffer AL, and purification using a DNeasy Mini spin column. Hydrolysis of a CAA-PBS solution at 100 °C for 30 min Online SPE: C18 guard column cartridge	Not reported	System: LC-QqQ Column: biphenyl column Eluent: 15% MeOH in H_2_O containing 0.1% FA.
[53], 2025	5mC, 5hmC, 5fC, 5caC	Blood samples	DNA Extraction: commercial kit, precipitated by adding isopropyl alcohol and NaCl. Purification: cold ethanol-H_2_O solution. Enzymatic Hydrolysis:automated platform by adds two successive enzyme mixes.	LOQ: 8.0 × 10−9mol/L for 5mC and 1.0 × 10−10 for 5hmC.	System: LC-Q TRAP Column: Method 1: Hypercarb Method 2: Force Biphenyl Eluent A: H_2_O + 0.1% acetic acid Eluent B: MeOH 0.1% acetic acid.

**Table 2:** Analyte quantification, sample pre-treatment, and instrument used in the reviewed papers.

Reference, Year	Sample	Instruments	MS Quantifications	Sample Pre-Treatment
[54], 2015	cH2AX and K5-acetylated H2AX	System: LC-QqQ Column: BEH C18, CSH C18 column Analysis method: MRM	Acetylated and phosphorilated h2ay	Acid Extraction: sulfuric acid Precipitation: trichloroacetic In-Solution Trypsin Digestion Desalting: C18 StageTip.
[55], 2015	human IMR90 fibroblast cells infected with adenovirus.	System: LC-Orbitrap Fusion/Q-Orbitrap Column: C18 Eluent A: H_2_O and 0.1% FA Eluent B: 95% ACN and 0.1% FA Analysis method: DDA	Histone PMTs	Acid Extraction: 0.2 M H_2_SO_4_ Precipitation: trichloroacetic acid Propionylation: propionic anhydride in 2-propanol pH 8 Digestion: trypsin Post-Digestion Propionylation Desalting: C18 material
[56], 2015	HEK293 T, HCT116 cells	System: LC-Q-Orbitrap Column: C18 Eluent A: FA in H_2_O Eluent B ACN Analysis method: full scan 2. Absolute Quantification System: LC-QTRAP Column: Agilent Zorbax 300 SB-C18 Eluent A: FA in H_2_O Eluent B: FA in ACN Analysis method: MRM	Homocysteinylation, methylation and acetylation on histone H3	Acid Extraction: hydrochloric acid Propionylation: NHS-propionate Digestion: trypsin. Post-Digestion Propionylation
[57], 2015	HeLa Cell Culture	System: LC-Orbitrap Column: C18 Eluent A: H_2_O Eluent B: ACN Analysis method: DDA	Histone peptides with modifications	Acidic Extraction: sulfuric acid Precipitation: trichloroacetic acid Propionylation: mixture of propionic anhydride and 2-propanol Digestion: trypsin Post-Digestion Propionylation Desalting: C18 Stage-tips
[58], 2015	frontal cortex from human donors with AD	Qualitative Analysis System: LC-QTOF Column: ProtID C18 nano-chip. Eluent A: H_2_O Eluent B: ACN Analysis method: DDA Quantitative Analysis System: LC-QqQ Column: Zorbax Eclipse Plus C18 Eluent A: FA in H_2_O Eluent B: FA in ACN Analysis method: MRM	Histone PTMs	Acid Extraction: sulfuric acid solution Precipitation: trichloroacetic acid Chemical derivatisation: reduction with dithiothreitol and iodoacetamide Digestion: trypsin
[59], 2015	HEK293T cells, PC9 cells, HeLa cells	System: LC-ion trap-Orbitrap Column: C18 Eluent A: H_2_O and FA Eluent B: FA and ACN. Analysis method: DDA	Histone PMTs	Acid Extraction: acid-based method. Method 1: Propionylation: propionic anhydride and isopropanol Digestion: trypsin Post-Digestion Propionylation: Desalting: C18-stage-tip Method 2: Propionylation: propionic anhydride and H_2_O Digestion: trypsin Labeling: phenyl isocyanate Acidification: trifluoroacetic acid Desalting: C18-stage-tips.
[60], 2015	HeLa S3 cell, Jurkat, HL60, cells, PANC cells and U2OS cells PC3 cells, LHCN M2, LHCN M2	1. Top-Down H2B Analysis (HeLa and Jurkat cells) System: LC-ion trap-Orbitrap 2. Top-Down H2B Analysis (cancer cell lines) System: LC-Orbitrap Fusion 3. Bottom-Up Histone Analysis System: LC-ion trap-Q-Orbitrap Column: C18 Eluent A: H_2_O with FA Eluent B: ACN with FA Analysis method: DDA	H2B isoform composition	Acid Extraction: sulfuric acid solution. Precipitation: Trichloroacetic acid Histone H2B Purification: RP-HPLC Propylanisation: Propionic Anhydride Digestion: trypsin for several hours. Post-Digestion Propionylation Desalting: C18-StageTips
[61], 2015	Normal B cells and B cells isolated from Peripheral blood CLL patients, CD19+ cells, M1–M4 cells, hTERT cells, RT4 cells a, T24 and UM-UC-3	System: LC-Q-TOF Column: C18 column Eluent A: trifluoroacetic acid in H_2_O Eluent B: trifluoroacetic acid in ACN Method analysis: DDA	Histone proteome	Acid Extraction: sulfuric acid solution Precipitation: trichloroacetic acid Gel Separation: SDS-PAGE In-Gel Digestion: trypsin,
[62], 2015	Human Neural Stem Cells	System: LC-ion trap-Orbitrap Column: C18 Eluent A: FA in H_2_O Eluent B: FA in ACN Analysis method: PRM	Histone modifications	Acid extraction: Precipitation: perchloric acid Propionylation: mixture of propionic anhydride and isopropanol. The pH is maintained at 8–9 using ammonium hydroxide Digestion: trypsin. Post-Digestion Propionylation Desalting: C18-stage-tips
[63], 2015	Human MCF7 breast cancer cells	System: LC-Q-Orbitrap Column: NanoEase C18 Eluent A: FA in H_2_O Eluent B: FA in ACN Analysis method: DIA	Histone PTMs	Acid extraction Chemical derivatisation: Propionylation: propionic anhydride. Digestion: trypsin
[64], 2015	hESCs strain WA09 (or H9)	System: Chip-LC-TOF-TOF-TOF Column: ChromXP C-18 chip Eluent A: FA in H_2_O Eluent B: FA in ACN Analysis method: SWATH	Histone PMTs	Acid Extraction: sulfuric acid Precipitation: trichloroacetic acid Propionylation: mixing propionic anhydride with 2-propanol Digestion: trypsin Desalting: C18 Stage-tips
[65], 2015	HeLa cells	System: LC-ion trap-Q-Orbitrap Column: homemade capillary column containing Jupiter C12 resin Eluent A: FA in H_2_O Eluent B: FA in ACN Analysis method: DDA	Histone PMTs	Acid Extraction: sulfuric acid. Precipitation: trichloroacetic acid Labeling: formaldehyde and cyanoborohydrate SDS-PAGE In-Gel Digestion: enzyme trypsin Second Labeling Desalting: ZipTip
[66], 2016	Karpas 422 cells	System: LC-Q-Orbitrap Column: Xselect HSS T3 C18 Mobile Phases: Eluent A: FA in H_2_O Eluent B: FA in ACN 2. Column: CORTECS HILIC Mobile Phases: Eluent A: FA and ammonium formate in H_2_O; Eluent B: FA and ammonium formate in a mixture of MeOH and ACN. Analysis method: MRM	Histone PMTs	Acid extraction: hydrochloric acid solution. Separation: HPLC Propionylation: propionate acid N-hydroxysuccinimide ester Digestion: enzyme trypsin. Post-Digestion Derivatization
[67], 2016	human primary monocyte derived macrophages	System: LC-QTOF Column: PepMap C18 Eluent A: 0.1% FA and 3% ACN Eluent B: 0.1% FA and 97% ACN. Analysis method: not reported	Histone PMTs	Acid extraction: 0.4 N sulfuric acid Precipitation: trichloroacetic acid Propionylation: 3:1 propionic anhydride:ACN in-solution digestion: trypsin
[68], 2016	human embryonic stem cells with and without retinoic acid (RA) stimulation	System: LC-Orbitrap Column: C18 column Eluent A: H_2_O and and 0.1% FA Eluent B: 95% ACN and 0.1% FA Analysis method: DIA, DDA	Histone PMTs	Acid Extraction: cold sulfuric acid solution Precipitation: trichloroacetic acid Propionylation: propionyl anhydride. Digestion: trypsin Post-Digestion Propionylation Desalting: Stage-tip, which contains a C18 material
[69], 2016	Nnormal MCF-10A cells, parental drug-sensitive MCF-7/WT cancer cells, drug-resistant MCF-7/ADR cancer cells	System: LC-QqQ Column: HILIC Eluent A: ammonium acetate Eluent B: ACN Analysis method: MRM	Asymmetric and symmetric dimethylated H3R2	Acid Extraction: sulfuric acid solution Precipitation: acetone Reduction: dithiothreitol and then alkylated with iodoacetamide. Digestion: enzyme thermolysin
[70], 2016	HCT-8, HCT-116 cell lines	System: LC-Q-Orbitrap Column: Acclaim PepMap RSLC Eluent A 0.1% FA in H_2_O Eluent B: 0.1% FA in 98% ACN Analysis method: DDA	Lysine-acetylome and global-phosphorylation	Chromatin Protein Extraction: urea solution Precipitation: trichloroacetic acid Reduction: dithiothreitol and then alkylated with indole-3-acetic acid. Digestion: trypsin Affinity Enrichment: anti-lysine-acetylation and anti-lysine-phosphorylation antibody beads.
[71], 2016	Human embryonic WA01 Oct4-eGFP knock-in reporter cell line	System: LC-Q-TOF Columns: C18 Eluent A: FA in H_2_O Eluent B: FA in ACN Analysis method: DDA	Histone PMTs	Acid extraction: acid not reported Precipitation: trichloroacetic acid Digestion: trypsin
[72], 2016	HeLa (ATCC CCL-2)	System: LC-Q-Orbitrap Columns: ReproSil-Pur C18 Eluent A: FA in H_2_O Eluent B: FA in a mixture of isopropanol and MeOH Analysis method: DDA	Acetylation changes on the histones peptide	Acidic histone Extraction: hydrochloric acid Propionylation: propionic anhydride. Digestion: trypsin, Desalting: UltraMicroSpin Column. Alternative Method: FASIL chemical modification: D3-acetylation directly on a filter. Desalting: C18 spin columns.
[73], 2016	serum samples of patients with acute myeloid leukemia, breast cancer, and nonsmall cell lung cancer	System: LC-ion trap-Q-Orbitrap Column: C18 Eluent: ACN in 0.125% FA Analysis method: DDA	Histone PMTs	Reduction and Alkylation: dithiothreitol and iodoacetimide. Digestion: trypsin-TPCK. Acidification: Trichloroacetic acid Desalting: SEP PAK Classic C18 column. Immunoprecipitation: Antibody Binding Second Digestion: trypsin Final Clean-up: C18 tips
[74], 2016	Fresh-frozen Human Breast Cancer Tissue	System: LC-Q-Orbitrap Column: in-house-made C18. Eluent A: FA in H_2_O Eluent B: FA in ACN Analysis method: DDA, full scan	Histone PMTs	Lysis and Digestion: buffer with SDS and an enzyme called Benzonase Gel Separation: SDS-PAGE gel. Chemical alkilation: D6-acetic anhydride. In-Gel Digestion: trypsin. Desalting: C18/C and SCX chromatography on StageTips.
[75], 2016	HeLa cells	System: LC-modern Orbitrap instrument Column: PepMap Easy-Spray Eluent A: FA in H_2_O Eluent B: FA in ACN Analysis method: MSX-DIA	Histone PMTs	Acid Extraction: sulfuric acid solution Precipitation: trichloroacetic acid Propionylation: mixture of propionic anhydride Digestion: trypsin Post-Digestion Propionylation Desalting: C18 Stage-tips
[76], 2017	Serum human samples	System: LC-Orbitrap Analysis method: DDA	Histone analysis untargeted	Protein Precipitation: trichloroacetic acid Acid Extraction: sulfuric acid Histone Precipitation: acetone Digestion: trypsin Desalting: ZipTip C18 columns
[77], 2017	HeLa cells and cells undergoing epithelial to mesenchymal transition (EMT)	1. Bottom-Up Analysis System: LC-Q-Orbitrap Column: ReproSil-Pur C18-AQ Eluent A: H_2_O and FA Eluent B: ACN and FA Analysis method: DIA 2. Middle-Down Analysis System: LC-Orbitrap Fusion Columns: Polycat A analytical	Histone H3 N-terminal tails	Acid Extraction: sulfuric acid solution Precipitation: trichloroacetic acid Bottom-Up MS: Digestion: trypsin Middle-Down MS: Digestion: GluC
[78], 2017	HCT116 colon carcinoma MCTS	1. System: LC-ion trap-Q-Orbitrap Column: Reprosil-Pur C18-AQ Eluent A: H_2_O and FA Eluent B: ACN and FA Analysis Method: DIA 2. Imaging Mass Spectrometry System: MALDI-TOF/TOF Laser: 800 laser shots per array position at a laser frequency of 1000 Hz. Resolution: Lateral resolution is either 75 μm or 35 μm.	Histone PMTs	Acid Extraction: sulfuric acid Precipitation: trichloroacetic acid Propionylation: propionic anhydride and ACN. Digestion: trypsin Post-Digestion Propionylatio Desalting: C18 Stage-tips Mass Spectrometry Analysis matrix (a-cyano-4-hydroxycinnamic acid, CHCA)
[79], 2018	Human CTCL cell line HuT78 from peripheral blood of patients with Sezary syndrome	System: LC-Modern Orbitrap instrument Columns: Acclaim PepMap RSLC C18. Eluent A: H_2_O and FA Eluent B: ACN and FA Analysis method: DDA	Modifications of the acetylated proteome.	Chemical reduction and alkylation Digestion: Lys-C/trypsin Labeling: tandem mass tags Clean-up: Oasis HLB cartridges Enrichment: anti-acetyl lysine antibody beaded agarose.
[80], 2018	HeLa,293T, human embryonic stem cells, andmyoblasts	System: LC-Orbitrap Column: Reprosil-Pur C18-AQ Eluent A: FA in H_2_O Eluent B: ACN and FA Method analysis: DIA	Histone PMTs	Acid Extraction: sulfuric acid Precipitation: trichloroacetic acid Propionylation: propionylation solution. Digestion: trypsin Post-Digestion Propionylation Desalting: C18 Stage-tips
[81], 2018	HeLa cells	Sistem: LC-Orbitrap Column: Reprosil-Pur C18-AQ nano-column Eluent A: FA in H_2_O Eluent B: ACN and FA Analysis method: DIA	Histone peptides	Acid Extraction: sulfuric acid solution. Precipitation: trichloroacetic acid TCA Propionylation: mixture of propionic anhydride Digestion: trypsin. Post-Digestion Propionylation Desalting: C18 Stage-tips
[82], 2018	HeLa S3 cells	System: LC-Fusion Lumos Orbitrap Column: in-house packed PolyCAT A WCX-HILIC) Eluent A: 75% ACN with 20 mM propionic acid (pH 6) Eluent B:75% H_2_O with FA (pH 2.5) Analysis method: DDA	Identification and localization of PTMs on histones	Acidic extraction: acid not reported Precipitation: trichloroacetic acid Digestion: enzyme GluC pH 4
[83], 2018	MCF7-A2 cell	System: LC-ion trap Method instrument details are not reported	Acetylation of the H3.3 histone variant	Method 1: In-Gel Digestion: trypsin. Method 2: In-Solution Digestion with Multiple Enzymes: trypsin, chymotrypsin, and GluC. Propionylation: propionic anhydride
[84], 2019	breast cancer cells (MDA-MB-468 and MDA-MB-453) treated with HDACi Panobinostat	System: LC-TOF-TOF-TOF Column: Triart C18 Eluent A: H_2_O in ACN Eluent B: ACN in FA Analysis Methods: DDA, SWATH	Targeted study of histones	Acid Extraction: hydrochloric acid solution Precipitation: trichloroacetic acid Propionylation: propionic anhydride solution Digestion: trypsin Post-Digestion Propionylation
[85], 2019	human embryonic kidney cells 293 (HEK293)	System: LC-Orbitrap Fusion Column: A Reprosil-Pur C18-AQ Eluent A: FA in H_2_O Eluent B: FA in ACN Analysis method: DIA	Histone PTM	Acid Extraction: sulfuric acid solution Precipitation: trichloroacetic acid Propionylation: propionic anhydride and ammonium hydroxide Digestion: trypsin Desalting: stage-tip made from C18 and Porous Graphitic Carbon resins.
[86], 2019	HeLa S3 cells	System: LC-Orbitrap Fusion Columns: different in-house packed columns: C18 (Reprosil-Pur) C30 (Develosil) PGC (Hypercarb) WCX-HILIC (PolyCAT A) Analysis method: Bottom-Up Analysis: DIA Middle-Down Analysis: full scan, DDA	Histone modifications	Acid Extraction: sulfuric acid solution Precipitation: trichloroacetic acid Bottom-up: Propionylation: propionic anhydride Digestion: trypsin Post-Digestion Propionylation Desalting: C18 stage-tips Middle-down HPLC Fractionation Digestion: GluC
[87], 2020	human myoblast cell line, LHCN-M2	DI-Orbitrap Fusion Tribrid Analysis method: SIM	Histone PTMs	Acid Extraction: cold sulfuric acid Precipitation: trichloroacetic acid Propionylation: condition not reported Digestion: Trypsin
[88], 2020	MKN45 cells. HeLa cells	Affi-BAMS Assay Workflow System: MALDI-TOF	Profiling of multiple proteins and PTMs	Affi-BAMS protocols The proteins are reduced with DTT and then alkylated with iodoacetamide Digestion: trypsin-TPCK. Desalting: SEP PAK Classic C18 columns Immunoprecipitation Bead Preparation: Specific antibodies conjugated to magnetic agarose beads overnight.
[89], 2020	cells culture	LC-TOF-TOF-TOF Column: Triart C18 Eluent: low pH reversed-phase gradient with FA and DMSO. Analysis method: DDA	Histone PMTs	Acid Extraction: sulfuric acid Precipitation: trichloroacetic acid Acetylation: acetic anhydride, hydroxylamine Propionylation: propionic anhydride. Digestion: trypsin Desalting: Sep-Pak C18 μElution Plate.
[90], 2020	peripheral blood mononuclear cells from human patients	System: LC-Orbitrap Column: in-house packed C18 Eluent A: 0.1%FA in H_2_O Eluent B: 0.1% FA in ACN Analysis method: targeted setup	Acetylation patterns of histone H4	Acid Extraction: Sulfuric acid Precipitation: trichloroacetic acid Gel Separation: Gel Electrophoresis Acetylation: d6-deuterated acetic anhydride. Digestion: trypsin Desalting: C18-StageTips
[91], 2020	HEK293T cells, KMS11 multiple myeloma cells and NSD2 selective knockout KMS11cells	1. System: LC-ion trap-Q-Orbitrap Column: A C18 Eluent A: FA in H_2_O Eluent B: FA in ACN 2. In-Depth Analysis with LC-Orbitrap Fusion Lumos Column: C18. Eluent A: FA in H_2_O Eluent B: FA in ACN Analysis Method: DDA	Histone PMT	Acidic extraction: hydrochloric acid Digestion: protease OmpT. Desalting: C18-zip-tip
[92], 2020	human breast cell lines: MCF-7/WT, MDA-MB-231 cells, MCF-10A	1. Non-Targeted Analysis System: LC-TOF-TOF-TOF Column: BioBasix SCX Eluent A: low-salt solution Eluent B:high-salt solution. 2. Targeted Analysis System: LC-QqQ Column: SB-C18 Eluent A: FA in H_2_O Eluent B: FA in ACN Analysis method: MRM	Site-specific histone methylations and acetylation assisted	Acid extraction: sulfuric acid solution. Precipitation: acetone. Reduction and alkylation: dithiothreitol and iodoacetamide. FASP Method: loading the solution into a filter unit with a 10 kDa cutoff. Digestion: trypsin
[93], 2020	HeLa-S3 cell, human bone marrow CD34+ cells from healthy donors (NBMs)	System: LC-QqQ Columns: PicoChip packed with Bischoff ProntoSIL C18-AQ resin Eluent A: FA in H_2_O Eluent B: FA in ACN Analysis method: MRM	Histone PMTs	Acid extraction: sulfuric acid Precipitation: Trichloroacetic Propionylation: mixture of isopropanol and propionic anhydride Digestion: trypsin. Post-Digestion Propionylation:
[94], 2021	KARPAS-422 cell line Z-138, MDA-MB-468, and Toledo lines	System: LC-Orbitrap Columns: C18 Eluent: mixture of ACN and FA. Analysis Methods: DDA, PRM	H3K27 and H4R3 methylation profiling	Histone Acid Extraction: sulfuric acid solution Precipitation: trichloroacetic acid Lysine Acetylation: acetic anhydride in ACN. Lysine Propionylation: propionic anhydride in ACN at pH 8. Digestion: Trypsin Desalting: Sep-Pak C18 μElution Plate
[95], 2021	Breast cancer cell line MDA-MB-468, frozen breast cancer and FFPE ovarian and head and neck cancers	System: LC-Q-Orbitrap Column: EASY-Spray Eluent A: FA in H_2_O Eluent B: FA in ACN Analysis method: DDA 2. Imaging System: MALDI-Q-Orbitrap Laser Settings:349 nm and 500 Hz. Pixel Size: spatial resolution of 35 x 35 μm.	Histone PMTs	Separation:SDS-PAGE gel. Chemical derivatisation: Acylation: D6-acetic anhydride or propionic anhydride In-gel digestion: trypsin. Post-Digestion Derivatization: propionic anhydride or phenyl isocyanate Desalting: StageTips before analysis. Preparation for MALDI-MS: indium-tin oxide slides and coated with a 2,5-dihydroxybenzoic acid matrix
[96], 2021	Chronic lymphocytic leukemia cell line MEC-1	System: LC-Modern Orbitrap instrument Columns: Acclaim Pepmap100 C18 Eluent A: FA in H_2_O Eluent B: FA in ACN Analysis method: DDA	Histone PMTs	Acid Extraction: sulfuric acid Precipitation: trichloroacetic acid. Chemical acylation: trimethylacetic anhydride. Digestion: trypsin Post-Digestion Derivatization Desalting: HyperSep SpinTip C18
[97], 2022	C3A hepatocytes (HepG2/C3A) cultured.	System: LC-HR-MS Column: C18 Eluent A: 2% ACN + 0.1% FA Eluent B: 80% ACN + 0.1% FA Analysis method: MS/MS detail not reported	Histone PMTs	Acidic histone Extraction: sulfuric acid Precipitation: cold solution of trichloroacetic acid Digestion: trypsin Propionylation: condition not reported Post digestion propionylation Desalting: HLB resin inside a well of a filter plate.
[98], 2022	human blood–derived monocytes	Sistem: LC-Q-Orbitrap Column: In-house packed columns Eluent A: H_2_O Eluent B: ACN Analysis method: full scan, PRM	Histone modifications	SDS-PAGE gel electrophoresis In-gel digestion: trypsin
[99], 2022	Lung adenocarcinoma and paracancerous tissue samples	System: LC-Q-Orbitrap Column: Acclaim PepMap RSLC Eluent A: FA in H_2_O Eluent B: FA in ACN Analysis method: DDA	Lysine acetylation and succinylation profile alterations	Precipitation: cold solution of trichloroacetic acid Reduction and alkylation: dithiothreitol and iodoacetamide Digestion: trypsin Desalting: Strata X C18 SPE column Labeling: tandem mass tags Desalting: Strata X C18 SPE column
[100], 2023	MCF7 cells line	System: MALDI-TOF-MS Laser Power: 20–40%. Laser Shots: 2000 shots per spot. Laser Frequency: 10,000 Hz.	PTM marks of H3 and H4 histones.	Affi-BAMS platform Digestion: enzyme LysC. Peptide Enrichment: affinity capture beads
[101], 2023	HEK293T	System: LC-Q-Orbitrap Columns: in-house packed ReproSil-Pur C18-AQ Eluent A: FA in H_2_O Eluent B: FA in ACN Analysis method: DIA	Histone propionylation	Acid Extraction: sulfuric acid Precipitation: trichloroacetic acid Propionylation: propionic anhydride at pH 8 using ammonium Digestion: Trypsin Post-Digestion Propionylation Desalting: StageTips
[102], 2023	HIV-infected and Meth exposed hMDMs	System: LC-QTRAP Column: Omega reversed-phase Eluent A: FA in H_2_O Eluent B: FA in ACN Analysis method: MRM	Histone H3 lysine 14 acetylation stoichiometry	Acid Extraction: chilled sulfuric acid solution. Precipitation: cold trichloroacetic acid Pre-Digestion Propionylation: propionic anhydride in ACN Digestion: trypsin. Post-Digestion Propionylation: Desalting: mixed cation exchange cartridges.
[103], 2023	human plasma	System: LC-Q-Orbitrap Column: 15 cm analytical column packed with ReproSil-Pur C18-AQ Eluent A: 0.1% formia acid in H_2_O Eluent B: 0.1% FA in ACN Analysis method: DDA	Histone PMTs	Nucleosome Immunoprecipitation: anti-histone H3.1 antibody Propionylation Digestion: trypsin.
[104], 2024	Human breast cancer cell lines, MCF-7 (luminal A), T47D (luminal A), BT474 (luminal B), ZR-75-30 (luminal B), HCC1954 (HER2+), SK-BR-3 (HER2+), MDA-MB-231. breast tissue (TNBC), and MDA-MB-468 (TNBC), normal breast cell line, MCF-10A,	System: LC-Modern Orbitrap instrument Column: A C18 column is used for separation. Eluent A: FA in H_2_O Eluent B: FA in ACN Analysis method: DDA Targeted Proteomics System: LC-Q-TRAP Column: InfinityLab Poroshell 120 SB C18 Analysis method: MRM	Histone PTMs	Acid Extraction: sulfuric acid Precipitation: Trichloroacetic acid Chemical Derivatization: Propionylation: condition not reported Digestion: trypsin Post-Digestion Propionylation: Desalting: C18 Pierce Spin Tips
[105], 2024	normal and tumor breast clinical samples	System: LC-Q-Orbitrap Column: EASY-Spray C18 Eluent A: FA in H_2_O, Eluent B ACN and FA. Analysis method: DDA	Histone PTMs and histone acylations: propionylations and butyrylations	Gel electrophoresis step Propionylation: propionic anhydride or deuterated propionic anhydride. Digestion: trypsin Post digestion derivatisation: deuterated propionic anhydride Acidification: trifluoroacetic acid Desalting: C18 StageTips
[106], 2024	HEK293 e 293T	System: LC-Q-Orbitrap Column: in-house fabricated C18 fused silica column Eluent A: FA in H_2_O Eluent B: FA in ACN Analysis method: DDA	Histone ubiquitination marks 2AK119ub and H2BK120ub.	Acid Extraction: sulfuric acid solution Precipitation: Trichloroacetic acid Digestion: trypsin Propionylation: propionic anhydride Desalting: C18 StageTips
[107], 2025	MEC-1 chronic lymphocytic leukemia cell line	System: LC-Modern Orbitrap instrument Column: Aurora C18 Eluent A: mixture of FA in H_2_O Eluent B:FA in 80% ACN Method analysis: DDA	Histone PMTs	Acid Extraction: ice-cold sulfuric acid Precipitation: ice-cold trichloroacetic acid Chemical Derivatization: trimethylammonium Digestion: enzyme Arg-C Ultra Post-Digestion Propionylation: trimethylammonium Desalting: AttractSPE^®^ Tips C18
[108], 2025	human lung adenocarcinoma cell line NCI-H1437	System: LC-Q-TOF Liquid Chromatography (LC) Column: Kinetex XB C18 Eluent A: FA in H_2_O Eluent B: FA in ACN Analysis method: SWATH DIA	Histone PMTs	Acid Extraction: sulfuric acid solution. Precipitation: trichloroacetic acid Propionylation: propionic anhydride Digestion: trypsin Post-Digestion Propionylation
[109], 2025	standard solutions	System: LC-Q-TOF Column: nanoEase M/Z Protein BEH C4 Column Eluent A: 0.1% FA in H_2_O Eluent B: 0.1% FA in ACN Analysis method: MRM	PMTs site localization, histone PMTs	No treatment for standard solutions

## Data Availability

The data that support the findings of this study are available from the corresponding author upon reasonable request.

## Abbreviations

The following abbreviations are used in this manuscript:

PMTs	post-translation modifications
Cyt	Cytidine
5mC	5-Methylcytosine
5hmC	5-Hydroxymethylcytosine
5caC	5-Carboxylcytosine
5fC	5-Formylcytosine
dC	Deoxycytidine
5mdC	5-Methyldeoxycytidine
5hmdC	5-Hydroxymethyldeoxycytidine
4mdC	N4-Methyldeoxycytidine
5cadC	5-Carboxyldeoxycytidine
5fdC	5-Formyldeoxycytidine
5-Aza-dC	5-Azadeoxycytidine
2o-mdC	2′-Oxymethylcytidine
dG	2′-Deoxyguanosine
5-fodC	5-Formyl-2′-deoxycytidine
5forC	5-Formylcytidine
5-fodU	5-Formyl-2′-deoxyuridine
5-forU	5-Formyluridine
5forCm	2′-O-methyl-5-formylcytidine
5-forUm	2′-O-methyl-5-formyluridine
5hmdU	5-Hydroxymethyldeoxyuridine
dA	2′-Deoxyadenosine
dT	Thymidine
N3-5gmdC	6-Azide-β-glucosyl-5-hydroxymethylcytidine
O6-Me-dG	O6-Methyl-2′-deoxyguanosine
O6-CM-dG	O6-Carboxymethyl-2′-deoxyguanosine
N6-CM-dA	N6-Carboxymethyl-2′-deoxyadenosine
εdA	N6-Vinyl-2′-deoxyadenosine
N2-Et-dG	N2-Ethyl-2′-deoxyguanosine
5-Cl-dC	5-Chloro-2′-deoxycytidine
Guo	Guanosine
Ado	Adenosine
Urd	Uridine
5-methyluridine	5-Methyluridine
dU	2′-Deoxyuridine
m6dA	N6-Methyl-2′-deoxyadenosine
3MeA	N3-Methyladenine
7MeG	N7-Methylguanine
6MeG	O6-Methylguanine
1MeG	1-Methylguanine
9MeG	9-Methylguanine
2EtdG	2-Ethyl-2′-deoxyguanosine
1MeA	1-Methyladenine
9MeA	9-Methyladenine
MeA	3-Methyladenine (come da tua lista)
9EtA	9-Ethyladenine
9EtG	9-Ethylguanine
Ino	Inosine
5-methyl-CMP	5-Methylcytidylic acid
FA	Formic Acid
ACN	Acetonitrile
MeOH	Methanol
LOD	Limit of Detection
LLOD	Lower Limit of Detection
LOQ	Limit of Quantification
LLOQ	Lower Limit of Quantification
RSD	Relative Standard Deviation
CV	Coefficient of Variation
LC	Liquid Chromatography
QqQ	Triple Quadrupole
Q	Quadrupole
TOF	Time-of-Flight
ICP	Inductively Coupled Plasma
MALDI	Matrix-Assisted Laser Desorption/Ionization
DESI	Desorption Electrospray Ionization
CE	Capillary Electrophoresis
HILIC	Hydrophilic Interaction Liquid Chromatography
LAMP	Linear Amplification
AD	Alzheimer’s Disease
CRC	Colon Rectal Cancer
Affi-BAMS	Affinity-bead Assisted Mass Spectrometry
SDS-PAGE	Sodium Dodecyl Sulfate Polyacrylamide Gel Electrophoresis
SPE	Solid Phase Extraction
DI	Direct Infusion
DDA	Data-Dependent Acquisition
DIA	Data-Independent Acquisition
SIM	Selected Ion Monitoring
PRM	Parallel Reaction Monitoring
MRM	Multiple Reaction Monitoring
SWATH	Sequential Window Acquisition of all Theoretical Fragmention Spectra
